# A Scoping Review of Digital Behavioral Approaches to Improve Medication Adherence in Type 2 Diabetes

**DOI:** 10.1007/s11892-026-01642-5

**Published:** 2026-09-05

**Authors:** Yen-Ming Huang, Pin-Heng Tiao, Olayinka Shiyanbola

**Affiliations:** 1 School of Pharmacy, College of Medicine, National Taiwan University, Taipei City, 100025 Taiwan; 2 Graduate Institute of Clinical Pharmacy, College of Medicine, National Taiwan University, Taipei City, 100025 Taiwan; 3 Department of Pharmacy, National Taiwan University Hospital, Taipei City, 100229 Taiwan; 4 Department of Clinical Pharmacy, College of Pharmacy, University of Michigan, Ann Arbor, MI 48109 USA

**Keywords:** Adherence, Behavioral, Digital, Intervention, Medication, Type 2 Diabetes

## Abstract

**Purpose of Review:**

Consistent medication adherence is fundamental for achieving favorable health outcomes in diabetes care; however, attaining optimal adherence remains a challenge. This review summarized recent evidence on digital behavioral interventions designed to improve medication adherence in type 2 diabetes (T2D) and identified key components commonly incorporated in these interventions.

**Recent Findings:**

Thirty-two studies evaluating digital strategies to support medication adherence were identified. Interventions were delivered through a range of digital platforms, including mobile apps, short message service messaging, telemedicine systems, conversational artificial intelligence, and remote monitoring technologies. Medication adherence was assessed using a variety of measures, including self-reported adherence questionnaires, medication-taking records, and refill-based measures. Most studies reported improvements in medication adherence, and several also observed improvements in glycemic outcomes, most commonly A1C. Effective interventions frequently combined reminders, educational content, self-monitoring tools, and patient-provider communication.

**Summary:**

Digital behavioral interventions represent increasingly utilized strategies to promote medication adherence among individuals with T2D. Multifaceted digital approaches that integrate behavioral support, monitoring, and communication may enhance long-term diabetes self-management and treatment engagement. Recent advances have focused on improving personalization, integration across digital platforms, and interactive patient support rather than introducing entirely new digital modalities.

**Supplementary Information:**

The online version contains supplementary material available at https://doi.org/10.1007/s11892-026-01642-5.

## Introduction

Optimal medication adherence is essential for achieving glycemic control and preventing long-term complications in individuals with type 2 diabetes (T2D) [1]. Despite the availability of effective pharmacologic therapies, many individuals experience challenges with maintaining medication use as prescribed during long-term diabetes management. These challenges may occur throughout different stages of medication use and are influenced by numerous individual, interpersonal, and healthcare system factors [2]. Challenges with medication adherence have been associated with increased risks of hospitalization, disease progression, and greater health care utilization [3]. The International Society for Pharmacoeconomics and Outcomes Research has estimated that approximately 33% to 69% of medication-related hospitalizations are attributable to difficulties with medication use and adherence, contributing to nearly US $100 billion in annual health care expenditures each year [4]. As a result, improving medication adherence continues to be recognized as a critical priority in diabetes management.

Medication adherence is a multidimensional behavior that extends beyond simply taking medications as prescribed. According to the widely adopted ABC taxonomy, adherence comprises three interrelated phases: initiation (starting a prescribed medication), implementation (the extent to which medication-taking corresponds with the prescribed regimen), and persistence (the duration of continued treatment before discontinuation) [5]. These phases reflect distinct aspects of medication-taking behavior and may be influenced by different behavioral, clinical, and contextual factors. Medication adherence can be evaluated using a variety of approaches, including self-reported adherence questionnaires, pharmacy refill records, electronic monitoring systems, medication possession measures, and clinical indicators [6]. Because each method captures different dimensions of adherence and varies in its sensitivity to behavioral change, recognizing adherence as a multidimensional construct is essential for interpreting the heterogeneous interventions and outcomes reported in the literature [7].

Medication-taking behaviors in T2D are affected by multiple interacting determinants that extend beyond clinical factors alone. Patients’ beliefs about treatment necessity, concerns about adverse effects, complexity of medication regimens, forgetfulness, and structural barriers related to medication access or health services can all influence adherence behaviors [8]. Challenges with medication adherence may arise from intentional decisions, such as when patients question the benefits of pharmacologic therapy or prefer to rely on lifestyle modification when the provider has recommended or prescribed a medication regimen [9]. In other situations, difficulties with medication-taking reflect unintentional barriers, including forgetfulness, competing daily priorities, or difficulties integrating medications into routine activities [10]. Because T2D requires lifelong pharmacologic management, effective adherence interventions must account for both the behavioral and contextual factors that influence medication-taking.

The factors influencing medication adherence extend beyond individual beliefs and behaviors to include broader social and structural conditions. Financial barriers, access to healthcare services, health literacy, housing instability, food insecurity, transportation challenges, work-related demands, caregiving responsibilities, and social support can all influence an individual’s ability to initiate, implement, and sustain medication use [11]. These factors may also contribute to disparities in treatment engagement and diabetes outcomes. Their importance becomes even more apparent in the context of digital adherence interventions, where access to smartphones, reliable internet connectivity, digital literacy, and ongoing engagement with healthcare services are often prerequisites for successful use [12]. As a result, social and structural conditions may influence not only medication adherence itself but also the reach, uptake, and effectiveness of digital health interventions. Recognizing these broader influences is important when interpreting intervention effectiveness and considering how digital adherence strategies can be implemented equitably across diverse populations.

In recent years, digital health technologies have received increasing attention as important tools to support medication adherence and chronic disease self-management [13]. Advances in mobile connectivity, wearable sensors, and telemedicine platforms have expanded opportunities to deliver behavioral support beyond traditional in-person care [14]. Mobile health interventions, including smartphone applications (apps), remote glucose monitoring systems, and telemedicine services, enable patients to receive education, reminders, and feedback in real time while allowing clinicians to monitor treatment progress remotely [15]. These technologies may reduce geographic and logistical barriers that limit access to health care services, particularly for individuals living in rural or medically underserved settings [16]. Digital platforms can also facilitate sustained engagement by integrating reminders, personalized feedback, and interactive communication between patients and healthcare professionals [17]. Because of their scalability and relatively low unit cost, digital interventions have generated considerable interest as potential strategies to improve treatment adherence and long-term disease outcomes [18]. A wide range of digital tools has been explored to support medication-taking behaviors in diabetes management [15, 19]. These include mobile apps that provide educational and medication reminders [20], telemedicine platforms that facilitate remote consultations [21], short message service (SMS) systems, automated phone calls, and web-based patient portals [22]. More recent innovations have incorporated additional features, such as support for insulin titration, video-assisted monitoring, and sensor technologies that help track medication use over time [23].

Although digital interventions for diabetes self-management have been evaluated in several prior reviews, most have focused on broad behavioral or clinical outcomes rather than medication adherence as a distinct therapeutic target [24–26]. Furthermore, the rapid evolution of digital health technologies since 2021, including remote patient monitoring, integrated digital platforms, conversational artificial intelligence (AI), and increasingly personalized approaches, has expanded both the range of available interventions and the ways in which they are delivered [14]. As these technologies continue to evolve, digital interventions have also become more complex, frequently combining medication reminders, educational content, self-monitoring, personalized feedback, and patient-provider communication within a single intervention [27]. This evolution has enabled more personalized and comprehensive adherence support. However, because many interventions now combine multiple components, it remains unclear which behavioral strategies contribute most to improved medication adherence [28]. As such, this scoping review synthesizes recent evidence on digital behavioral interventions designed to improve medication adherence in people with T2D by mapping intervention characteristics, identifying their behavioral components, summarizing approaches used to assess medication adherence, and describing reported glycemic outcomes. By providing an updated overview of contemporary digital adherence interventions, this review aims to inform future research and support the development of more effective and patient-centered strategies to improve medication adherence in diabetes care.

## Methods

A scoping review methodology was selected because of the diversity in study designs, intervention approaches, and outcome measures in this field [29]. The review followed the framework proposed by Arksey and O’Malley, which includes identifying the research question, identifying relevant studies, study selection, data charting, and summarizing the results [30]. Reporting of the review was guided by the Preferred Reporting Items for Systematic Reviews and Meta Analyses Extension for Scoping Reviews to enhance transparency and reproducibility [31]. A review protocol was developed a priori to guide the review process, including the research question, eligibility criteria, search strategy, data extraction procedures, and analytic approach. The protocol was used internally by the research team to ensure methodological consistency but was not formally registered or made publicly available.

### Identifying the Research Questions

The research question was structured using the Population, Concept, and Context framework, which is commonly applied in scoping reviews synthesizing health services and health promotion research [32]. The population included individuals with T2D. The concept focused on digital technology driven educational and/or behavioral interventions aimed at improving medication adherence. The context included clinical and/or community settings in which such interventions were implemented (Table 1). The review aimed to identify recent digital interventions targeting medication adherence and to map the key components commonly used within these programs.

**Table 1:** Research question development and study eligibility criteria guided by the Population-Concept-Context (PCC) framework

PCC tool	Inclusion criteria	Exclusion criteria
P – Population/Problem	• People with type 2 diabetes	• Caregivers • Healthcare professionals
C - Concept	• Core components of intervention or educational strategies intended to support better medication adherence • Use of digital technology-driven educational or behavioral interventions to improve medication adherence, defined as the initiation, implementation, and persistence of prescribed diabetes medications	• Absence of digital technology-driven educational or behavioral interventions to improve medication adherence • Studies focusing exclusively on diet, physical activity, foot care, or glycemic control, without addressing medication adherence
C - Context	• Use of digital technologies in clinical or community settings	• Emergency or urgent care settings
Type of studies	• Qualitative, quantitative, or mixed methods studies • Observational and experimental, cross-sectional, or longitudinal, randomized controlled trial or nonrandomized or noncontrolled trial, case series or case reports	• Review, meta-analysis, protocol, conference paper, editorials, letters, notes, book chapters, erratum or retraction
Language	• English	• Language other than English
Publication date	• From January 2021 to 2025 December	

### Information Sources and Search Strategy

A literature search was conducted in PubMed, Scopus, and Web of Science to identify relevant studies published between January 2021 and December 2025. The search strategy combined terms related to T2D, medication adherence, education, and behavioral or digital interventions. Keywords included “type 2 diabetes,” “medication adherence,” “medicine adherence,” “education,” and “intervention,” which were combined using Boolean operators. Searches were limited to titles and abstracts to improve specificity. All records identified through the database searches were exported to EndNote for reference management and removal of duplicate records. The full search strategies are provided in Appendix 1.

### Study Selection

Titles and abstracts were screened using predefined eligibility criteria (Table 1) by one reviewer and independently verified by a second reviewer. Full text articles were then assessed independently by two reviewers. Any disagreements during either stage of study selection were resolved through discussion and consensus, with consultation from a third reviewer when necessary. Studies were eligible if they evaluated educational and/or behavioral interventions designed to improve medication adherence among individuals with T2D and were published in English during the predefined study period. Medication adherence was required to be an explicit intervention target, outcome, or evaluation component. Broader diabetes self-management interventions were also eligible if medication adherence was assessed as either a primary or secondary outcome. Studies that focused exclusively on other self-management, such as diet, physical activity, foot care, or glycemic control, without evaluating medication adherence were excluded.

### Data Charting and Synthesis

Data were charted using a predefined extraction form developed with reference to previous literature [33–35]. Extracted data included publication year, country, study design, participant characteristics, intervention components, and reported outcomes related to medication adherence or glycemic control. Because medication adherence was the primary focus of this review, information regarding adherence assessment methods was also extracted. Eligible studies measured adherence using a variety of approaches, including self-reported adherence questionnaires or scales, medication-taking records, refill-based measures, medication use logs, and intervention-specific adherence indicators. When reported, objective clinical outcomes associated with adherence, such as A1C or fasting plasma glucose, were also extracted. Data extraction was conducted by one reviewer and cross verified by other members of the research team to ensure accuracy. All extracted data were organized using Microsoft Excel.

A descriptive synthesis approach was undertaken to summarize study characteristics and intervention components. Frequencies and percentages were calculated to describe study design, geographic distribution, and intervention characteristics. To identify common behavioral approaches used to improve medication adherence, intervention descriptions were analyzed using inductive content analysis [36]. Intervention components were coded inductively according to their primary function and categorized into common behavioral strategies, including medication reminders, educational content, self-monitoring, patient-provider communication, peer support, telemonitoring, and personalized feedback.

Consistent with the objectives of scoping reviews, the purpose of this review was to map the breadth and characteristics of the available evidence rather than to evaluate intervention effectiveness or determine comparative efficacy. Therefore, a formal risk-of-bias or methodological quality assessment was not conducted. This approach aligns with established scoping review methodology and should be considered when interpreting findings related to intervention effectiveness, as the included studies may vary in methodological rigor [32].

## Results

### Search Results

**Fig. 1:**
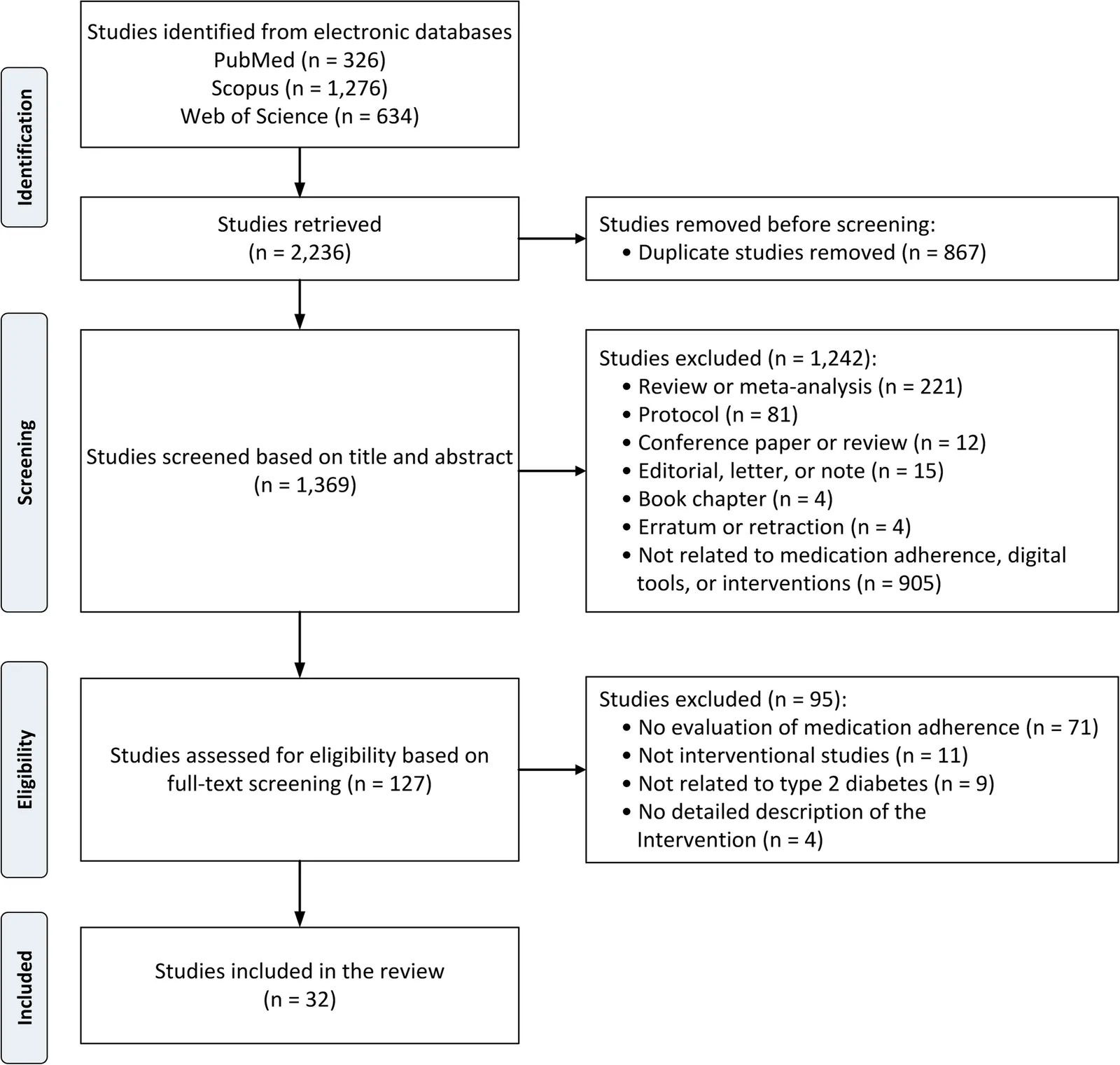
Flow diagram of the literature search process

The initial search identified 2,236 records, of which 867 were duplicates. After screening titles and abstracts, 1,242 articles were excluded, leaving 127 studies for full text assessment. Of these, 71 were excluded due to lack of assessment of medication adherence, 11 were non-interventional studies, 9 did not involve individuals with type 2 diabetes, and 4 lacked sufficient detail on intervention components. Ultimately, 32 studies [4, 16, 18, 37, 39–42, 45–51, 53–56, 58–68] were included in the qualitative synthesis. Figure 1 illustrates the study selection process.

### Study Characteristics

The included studies involved diverse populations and were conducted across multiple regions. Most studies were carried out in the United States (*n* = 10, 31.3%) [4, 38, 41, 44–48, 52, 62], followed by Spain (*n* = 4, 12.5%) [18, 39, 57, 65]. Additional studies were conducted in Asia (*n* = 14, 43.8%) [40, 42, 46, 48–51, 53, 55, 58, 61, 63, 64], Africa (*n* = 2, 6.3%) [16, 37], Australia (*n* = 2, 6.3%) [49, 59], the Middle East (*n* = 2, 6.3%) [56, 60], and the United Kingdom (*n* = 1, 3.1%) [43]. Most studies used randomized controlled trial designs (*n* = 27, 84.4%) [16, 18, 37, 38, 42–52, 54–65]. Four studies (12.5%) used a single-group pre–posttest design [39, 41, 53], and one study (3.1%) used a case-crossover design [4]. Sample sizes ranged from 13 to 1,119 participants. All studies included participants of both sexes, except for one study that enrolled only male veterans [4]. In addition, 43.8% of the studies reported a majority of male participants [4, 18, 43, 45, 48, 49, 51, 53, 55, 58–60, 64, 65] (Table 2).

**Table 2:** Overview of study characteristics, intervention components, and adherence and glycemic outcomes (*n* = 32)

No	Author(s), Year	Country	Study Design	Sample size/treatment assignment	Mean age (SD)	Sex	Participant characteristics	Intervention components	Adherence/glycemic outcomes
1	Farmer et al., 2021 [37]	South Africa and Malawi	Two-arm randomized controlled trial	C: 561, usual care I: 558, usual care and automated text message support	57.1 (11.4)	C: 392 (69.9%) female I: 390 (69.9%) female	Adults with T2D receiving at least one oral diabetes medication	3–4 automated motivational and educational text messages sent to patients each week	MA: *p* = 0.075 A1C: *p* = 0.537
2	Nelson et al., 2021 [38]	United States	Two-arm randomized controlled trial	C: 253, usual care I: 253, mobile phone-delivered interventions	C: 55.8 (9.8) I: 56.1 (9.4)	C: 139 (54.9%) female I: 135 (53.4%) female	Patients with T2D and prescribed one or more daily diabetes medications	(1) one-way self-care promotion texts, (2) interactive texts assessing medication adherence, (3) adherence feedback texts providing weekly feedback and encouragement based on responses to the interactive texts	MA: *p* = 0.003 A1C: non-significant
3	Roca et al., 2021 [39]	Spain	Single-group pre-post test design	13	63.8 (9.1)	9 (69.2%) female	Patients with T2D and depression disorder	The virtual assistant presents options, and the patient responds by entering the corresponding number or typing the selected option as text	MA: *p* = 0.004 A1C: *p* = 0.02
4	Umaya et al., 2021 [40]	Indonesia	Single-group pre-post test design	30	NA	NA	Patients with T2D	Medication reminder application	MA: *p* < 0.001
5	Wilson-Anumudu et al., 2021 [41]	United States	Single-group pre-post test design	195	45.1 (8.9)	136 (69.7%) female	Adults with T2D and A1C of 7% or greater	Remote monitoring and lifestyle change, in addition to comprehensive diabetes education	MA: *p* = 0.01 A1C: *p* < 0.001
6	Wungrath et al., 2021 [42]	Thailand	Two-arm randomized controlled trial	C: 30, usual care I: 30, Line group activities and telephone-based counseling	C: 59.2 (13.5) I: 61.8 (11.2)	C: 20 (66.7%) female I: 17 (56.7%) female	Adults with uncontrolled T2D	Line group activities (5-7-minute videos covered the 5Rs: right medicine, dose, route, patient, and time) and telephone-based counseling (5–10 min counseling sessions in weeks 3, 5, and 7)	MA: *p* < 0.001
7	Bartlett et al., 2022 [43]	United Kingdom	Two-arm randomized controlled trial	C: 106), 1 message monthly I: 103, 4 messages monthly	C: 63.4 (9.7) I: 63.5 (10.6)	C: 62 (58.5%) male I: 61 (59.2%) male	Adults of 35 years or older with T2D on oral diabetes medication	4 weekly texts for 6 months promoting medication adherence with validated behavior change techniques and supporting diet and physical activity	MA: *p* = 0.001
8	Mahoney et al., 2022 [44]	United States	Two-arm randomized controlled trial	C: 30, usual care I: 28, diabetes care application and new pen needle	C: 59.4 (10.8) I: 57.0 (9.8)	C: 18 (60.0%) male I: 19 (67.9%) female	Adults of 22 years or older with T2D on multiple daily insulin injections and A1C 8–11%	3–5 automated motivational and educational text messages sent to patients each week	MA: *p* = 0.46 FPG: non-significant
9	Olomu et al., 2022 [45]	United States	Two-arm randomized controlled trial	C: 26, mHealth only I: 25, mHealth and Office-Guidelines Applied to Practice	C: 60.6 (12.6) I: 54.3 (12.6)	C: 17 (65.4%) male I: 13 (52.0%) male	Adults with T2D and A1C of 8% or greater	Patient activation group visit, provider training, real-time decision support checklist used during the clinical encounter, and daily text messages for 15 weeks	MA: non-significant
10	Pamungkas et al., 2022 [46]	Indonesia	Two-arm randomized controlled trial	C: 30, usual care I: 30, smartphone application of diabetes coaching intervention	C: 54.5 (9.2) I: 56.2 (7.6)	C: 19 (63.3%) female I: 24 (80.0%) female	Adults aged 35–59 years with T2D and A1C of 7% or greater	Narrative App-based coaching, a printed user guide, mindfulness-based coaching, skill-based coaching, and a small App-interaction	MA: *p* < 0.001 A1C: *p* < 0.001
11	Poonprapai et al., 2022 [47]	Thailand	Two-arm randomized controlled trial	C: 79, usual care I: 78, smartphone application of diabetes coaching intervention	C: 67.8 (6.2) I: 67.4 (5.7)	C: 47 (59.5%) female I: 47 (60.3%) female	Adults of 65 years or older with T2D, A1C greater than 7%, and on oral diabetes medication	3–5 daily infographics via a mobile app for 3 months, covering diabetes knowledge, self-management, medications, comorbidities, caregiver motivation, and reminders for medication and appointments	MA: *p* < 0.001 A1C: *p* < 0.001
12	Zhou et al., 2022 [48]	China	Two-arm randomized controlled trial	C: 47, usual care I: 47, Internet+ Intelligent 5 A care model	C: 67.6 (4.3) I: 67.7 (4.4)	C: 25 (53.2%) male I: 26 (55.3%) male	Adults aged over 60 years with T2D	Interdisciplinary team, digital platform support, patient assessment via questionnaires, targeted guidance, one-to-one help, and follow-up through QQ, WeChat, and cloud groups to improve self-care and adherence	MA: *p* < 0.01 A1C: *p* < 0.05
13	Cheung et al., 2023 [49]	Australia	Two-arm randomized controlled trial	C: 448, usual care I: 454, tailored text message support	C: 61.9 (12.0) I: 61.1 (11.1)	C: 320 (71.4%) male I: 325 (71.6%) male	Adults with T2D and A1C 7.1–11.4%	C: standard care with administrative SMS text messages only I: standard care supplemented with four semipersonalized SMS text messages per week providing self-management support	MA: *p* = 0.045 A1C: *p* = 0.35
14	Hoda et al., 2023 [50]	India	Two-arm randomized controlled trial	C: 50, usual care I: 50, usual care combined with text messages and telephonic calls	C: 52.3 (10.1) I: 49.8 (8.6)	C: 27 (54.0%) female I: 31 (62.0%) female	Adults with T2D and A1C of 7% or greater	3 to 5 text messages per week and a weekly telephonic consultation	MA: *p* < 0.001 A1C: *p* < 0.001
15	Mangaraj et al., 2023 [51]	India	Two-arm randomized controlled trial	C: 60, usual care I: 140, telemedicine-based diabetes care	C: most (50.0%) between 25–34 years I: most (46.4%) between 25–34 years	C: 32 (53.3%) male I: 74 (52.9%) male	Adults with T2D	Comprehensive telemedicine-based diabetes care, including virtual consultations with healthcare providers, medication reminders via mobile apps, and continuous digital health monitoring of glucose levels, physical activity, and medication adherence	MA: *p* < 0.001 A1C: *p* < 0.001
16	Nayak et al., 2023 [52]	United States	Two-arm randomized controlled trial	C: 16, usual care I: 16, voice-based conversational artificial intelligence application	C: 56.2 (13.5) I: 54.1 (12.3)	C: 10 (62.5%) female I: 9 (56.3%) female	Adults with T2D and required initiation or adjustment of once-daily basal insulin	Voice-based conversational artificial intelligence application	MA: *p* = 0.01 A1C: *p* = 0.005
17	Rai et al., 2023 [4]	United States	Case-crossover design	30	Median: 66	100% male	Veterans with T2D and poor A1C	Novel smartphone app (DayaMed Arthur; DayaMedicals Incorporated) delivering medication reminders and caregiver notifications based on participants’ current regimens	MA: *p* = 0.02 A1C: *p* = 0.30
18	Sawaengsri et al., 2023 [53]	Thailand	Single-group pre-post test design	29	60.5 (7.7)	21 (72.4%) male	Adults aged over 35 years with T2D, A1C greater than 7%, and on oral diabetes medication	Usual care provided during the first 4 weeks, followed by usual care combined with brief motivational interviewing during weeks 4–8	MA: *p* < 0.001 A1C: non-significant
19	Zamanillo-Campos et al., 2023 [18]	Spain	Two-arm randomized controlled trial	C: 111, usual care I: 96, usual care and text message	C: 61 (12) I: 63 (10)	C: 74 (66.7%) male I: 61(63.5%) male	Adults with T2D and A1C greater than 8%, and on at least one oral diabetes medication	5 messages per week were delivered addressing key areas of diabetes self-management, including healthy diet, physical activity, and diabetes medication, while also providing general information about T2D	MA: *p* = 0.053 A1C: *p* = 0.670
20	Shaban et al., 2024 [16]	Egypt	Two-group pretest–posttest design	C: 60, usual care I: 60, digital based nursing intervention	C: 57.1 (7.0) I: 55.3 (3.2)	C: 30 (50%) female I: 32 (53.3%) female	Adults with T2D and basic computer literacy	A diabetes self-management mobile application providing personalized education on self-care, medication adherence, diet, and physical activity, along with real-time glucose monitoring, self-assessment quizzes, and peer support	MA: *p* = 0.027
21	Verma et al., 2024 [54]	India	Three-arm randomized controlled trial	C: 30, standard care I 1: 30, standard care and pillbox I 2: 30, standard care and short message service	NA	NA	Adults with T2D and prescribed diabetes medications	SMS and pillbox interventions	MA: *p* < 0.001 A1C: *p* = 0.04
22	Ye et al., 2024 [55]	China	Two-arm randomized controlled trial	C: 77, usual care I: 78, usual care and online education	C: 58.2 (5.1) I: 57.0 (3.8)	C: 45 (58.4%) male I: 41 (52.6%) male	Adults aged 30–60 years with T2D and hypertension	Online education through the WeChat application	MA: *p* = 0.01 A1C: *p* = 0.048
23	Bakhtiar et al., 2025 [56]	Iran	Two-arm randomized controlled trial	C: 30, usual care I: 30, mobile app delivering audio alerts and a cartoon animation	C: 59.2 (2.2) I: 53.7 (2.1)	C: 16 (53.3%) male I: 16 (53.3%) female	Adults aged 18–70 years with T2D, A1C of 5.7% or greater, and on at least one oral diabetes medication	Reminder of the time to take medicines, a cartoon animation, and nine educational questions	MA: *p* < 0.01 A1C: *p* = 0.35
24	Caballero Mateos et al., 2025 [57]	Spain	Two-arm randomized controlled trial	C: 41, usual care I: 44, digital intervention	C: 54.2 (12.9) I: 53.0 (10.9)	C: 21 (51.2%) female I: 28 (63.6%) female	Adults with T2D, A1C greater than 7, and initiating glucagon-like peptide-1 receptor agonist	Education in using social networks and diabetes self-care and an online therapeutic education specialist (Digital Diabetes Coach)	MA: *p* = 0.01 A1C: *p* = 0.006
25	Chen et al., 2025 [58]	China	Two-group pretest–posttest design	C: 68, standard nursing interventions I: 52, “Internet+” integrated continuous nursing model based on the Omaha System	C: 51.9 (7.3) I: 53.1 (6.8)	C: 37 (54.4%) male I: 31 (59.6%) male	Adults with T2D	Online health management platform, establishing an online health management team, and implementing continuous nursing care utilizing the Omaha System	MA: *p* = 0.93 FPG: *p* < 0.05
26	Höld et al., 2025 [59]	Austria	Two-arm randomized controlled trial	C: 27, usual care I: 41, peer support instant messaging service	C: 61.1 (8.9) I: 62.0 (8.5)	C: 27 (65.9%) male I: 20 (74.1%) male	Patients with T2D and A1C of 6.5% or greater	Peer support instant messaging service	MA: *p* > 0.05 A1C: *p* > 0.05
27	Hosseini et al., 2025 [60]	Iran	Two-arm randomized controlled trial	C: 47, usual care I: 49, usual care combined with educational videos and booklets on diabetes mellitus	C: 57.4 (14.6) I: 55.4 (17.6)	C: 27 (57.4%) male I: 26 (53.1%) female	Adults with T2D	Educational videos and booklets about the correct method of insulin injection and its importance across three online sessions	MA: *p* = 0.002
28	Lee et al., 2025 [61]	Singapore	Two-arm randomized controlled trial	C: 156, usual care I: 159, home tele-monitoring	C: 53.3 (7.8) I: 52.0 (7.4)	C: 91 (56.9%) female I: 97 (62.2%) female	Adults (26–65 year) with T2D and A1C 7–10%	Tele-education, tele-monitoring, and tele-support	MA: *p* = 0.146 A1C: *p* < 0.001
29	Pekmezaris et al., 2025 [62]	United States	Two-arm randomized controlled trial	C: 120, comprehensive outpatient management I: 120, diabetes telehealth management	C: 55.3 I: 56.1	C: 76 (63.3%) female I: 81 (67.5%) female	Hispanic/Latino adults with T2D	Daily self-monitoring of vital signs, weekly telehealth visits with a nurse, and eight educational videos	MA: *p* = 0.18 A1C: *p* < 0.01
30	Tan et al., 2025 [63]	Singapore	Two-arm randomized controlled trial	C: 160, usual care I: 159, home tele-monitoring	C: 53.3 (7.8) I: 52.0 (0.6)	C: 91 (56.9%) female I: 98 (61.6%) female	Adults (26–65 year) with T2D and A1C 7–10%	Tele-education, tele-monitoring, and tele-support	MA: *p* = 0.691 A1C: *p* < 0.001
31	Yu et al., 2025 [64]	Republic of Korea	Two-arm randomized controlled trial	C: 30, face-to-face education I: 26, face-to-face education and remote monitoring	C: 61.3 (7.3) I: 59.0 (10.8)	C: 22 (84.6%) male I: 20 (66.7%) male	Adults of 19 years or older with T2D and A1C 7.5–9.5%	Face-to-face education delivered through the digital platform at enrollment and follow-up visits, accompanied by remote monitoring for the initial 3 months	MA: *p* = 0.59 A1C: *p* = 0.01
32	Zamanillo-Campos et al., 2025 [65]	Spain	Two-arm randomized controlled trial	C: 371, usual care I: 371, tailored text message	C: 65 (10) I: 65 (10)	C: 217 (58.5%) male I: 216 (58.2%) male	Adults with T2D and A1C greater than 7.5%, and on at least one oral diabetes medication	5 SMSs per week for 4 months, three SMSs per week for the next 4 months, and 2 SMSs per week for the last 4 months	MA: *p* = 0.033 A1C: *p* = 0.772

*C* Control group, *FPG* Fasting plasma glucose, *I* Intervention group, *MA* Medication adherence, *NA* Not available, *SD* Standard deviation, *SMS* Short message service, *T2D* Type 2 diabetes

### Digital Intervention Components

The digital interventions were categorized according to both the digital platform used and the behavioral components incorporated within the intervention. The digital health tools identified in this review included mobile apps (delivered via smartphones, tablets, personal computers, or personal digital assistants), voice-based conversational artificial intelligence (AI), cloud-based platforms, remote patient monitoring systems, telephone-based interventions, and SMS text messaging. Applications were further classified into two categories, including diabetes-specific software (e.g., DayaMed Arthur, Doctorvice, OPTIMUM) and instant messaging software (e.g., WeChat, WhatsApp, and LINE). Among the included studies, eight studies [18, 37, 38, 43, 45, 49, 54, 65] delivered interventions exclusively through SMS text messaging, 1 study [53] used telephone-based communication only, 1 study [52] used voice-based conversational AI only, and 12 studies [4, 16, 39, 40, 56, 58, 63–66] used diabetes-specific software as the sole intervention platform. Ten studies [41, 42, 46–48, 50, 51, 55, 57, 64] implemented two or more digital health tools as part of the intervention.

The functions and content of app-based interventions were categorized into several key components, including adaptive, personalized reminders and alerts, educational hubs for diabetes knowledge, integrated monitoring systems, patient-healthcare professional support systems, and social support systems. Voice-based conversational AI primarily provided interactive self-management support, while cloud-based platforms mainly facilitated educational content delivery and patient-healthcare professional communication. Remote patient monitoring systems focused on continuous health monitoring and data integration. SMS-based interventions typically delivered motivational messages and tailored health education related to medication adherence and healthy lifestyle behaviors. Detailed descriptions of the digital health tools and their functions are presented in Table 3.

**Table 3:** Types, functions, and intervention components of digital health tools in diabetes interventions

Classification of digital health	number	Function and intervention contents
Application	21	
Diabetes-specific software [4, 16, 39–42, 44, 46–48, 51, 55–64]	21	• Adaptive, personalized reminders and alerts –Treatment reminder: follow-up appointments –Medication reminder: medication time, dosage, and drug interaction • Intelligent educational hub –Basic medication knowledge: right medication, dose, route, name, and time through video clips –Diabetes knowledge: disease management, lifestyle recommendations, pharmacological therapy delivered through animated videos, infographics, posts, and interactive self-assessment quizzes –Skills of medication administration: insulin injection technique • An integrated monitoring system –Manually entered information: self-management activities –Real-time glucose monitoring • Patient–healthcare professional support system –One-on-one guidance to support emotional well-being, enhance self-care skills, and address gaps in understanding –Virtual consultations with healthcare professionals • Social support system –Peer support forum for group discussions
Instant messaging software [46–48, 55, 59]	3	• Psychological intervention: mindfulness-based coaching (empowering messages) • Instant interaction: answering questions related to diabetes self-management, online consultations • Instant messaging-based peer support for discussion and exchange
Voice-based conversational AI [52]	1	• Intelligent self-management support –Information on medication use: insulin dosing and timing –Diabetes management: blood glucose monitoring and target goals
Cloud-based platform [48, 57]	2	• Intelligent educational hub –Basic diabetes education: disease management, lifestyle recommendations, and medication therapy • Patient-healthcare professional communication –Supporting questions on diabetes self-management
A remote patient monitoring system [41, 51]	2	• Intelligent monitoring hub –Multidimensional data collection: real-time access to patient physiological indicators (e.g., body, blood sugar) –Continuous health monitoring: tracking glucose levels, physical activity, and medication adherence
Telephones [41, 42, 46, 50, 53, 57]	6	• Reduction of prescription errors • Supplementary support regarding medication adherence issues • Tracking diabetes self-management practices, monitoring potential complications, and addressing barriers to program implementation • Tailored/general health education on medication-taking, healthy lifestyle management (e.g., diet, exercise, smoking), and blood glucose self-monitoring
SMS test messaging [18, 37, 38, 43, 45, 47, 49, 50, 54, 64, 65]	11	• Positive motivational text messages • Tailored/general health education about medication-taking and healthy lifestyle (e.g., diet, exercise, self-monitoring of blood glucose) • Interactive texts about medication adherence • Regular tailored feedback on mediation adherence • Regular reminders for medication intake

Across the included studies, several recurring behavioral strategies were identified regardless of the technology platform. Medication reminders and alerts were the most frequently incorporated intervention component and were commonly delivered through SMS messages, mobile applications, telephone systems, or automated digital platforms. Educational content was another prominent feature and was provided through videos, infographics, interactive learning modules, or digital coaching. Many interventions also incorporated self-monitoring functions that enabled patients to record medication use, blood glucose levels, or other diabetes-related indicators. Patient-provider communication was frequently facilitated through secure messaging, teleconsultations, or digital coaching platforms, while several interventions incorporated peer-support or social networking features. Personalized feedback, often generated from patient-reported data or monitoring results, was another commonly identified strategy.

### Assessment of Medication Adherence

Medication adherence was assessed using a variety of methods across the included studies. Self-reported measures were the most frequently used (*n* = 29) [4, 16, 18, 37, 38, 40–46, 48–51, 53–65], including validated adherence questionnaires, medication-taking scales, and intervention-specific adherence assessments. Objective measures were used less frequently. Two studies assessed medication-taking behavior using pill counts [47] or medication use logs [52], while three studies evaluated adherence indirectly through pharmacy refill records using the medication possession ratio [4, 39, 65].

Most interventions targeted adherence to overall diabetes medication regimens rather than specific medication classes. A small number of studies focused on particular therapies, including oral antidiabetic medications, insulin titration programs, or glucagon-like peptide-1 receptor agonist treatment support. The diversity of adherence measures and medication targets limited direct comparisons across medication classes, and no clear patterns in intervention effectiveness according to medication type were identified.

### Impact of Digital Interventions on Medication Adherence and Glycemic Outcomes

Although studies were not selected based on the assessment of glycemic outcomes and primarily focused on medication adherence, glycemic indicators were extracted when reported. Twenty-one studies (65.6%) [16, 38–43, 46–57, 60, 65] reported statistically significant improvements in medication adherence following digital interventions. Glycemic outcomes were reported in 26 studies (81.2%) [4, 18, 37, 39, 41, 46, 49–51, 53–56, 58–62, 65–68]. Of these, most (*n* = 24) [4, 18, 37, 39, 41, 46, 49–51, 53–56, 58–62, 65–68] assessed A1C levels, while two studies [36, 50] measured fasting plasma glucose. Sixteen studies (61.5%) [39, 41, 46–48, 50–52, 54, 55, 57, 58, 61–64] showed statistically significant improvements in glycemic control following digital interventions. Of note, 11 studies [39, 41, 46–48, 50–52, 54, 55, 57] demonstrated concurrent improvements in both medication adherence and glycemic outcomes after implementation of digital interventions.

Several common characteristics were observed among interventions reporting significant improvements in medication adherence. Successful interventions were more likely to combine multiple behavioral strategies rather than relying on a single digital function (Table 1). Medication reminders were frequently integrated with educational content, self-monitoring tools, and opportunities for communication with healthcare professionals. Personalized feedback based on patient-reported information or monitoring data was also commonly incorporated in interventions demonstrating improvements in both medication adherence and glycemic outcomes. In contrast, interventions relying primarily on one-way reminder systems or educational messaging alone generally reported more variable findings. Although formal comparisons between intervention types were beyond the scope of this review, these observations suggest that integrating multiple behavioral strategies may provide greater support for medication adherence than single-component interventions.

## Discussion

This scoping review mapped the characteristics and components of digital technology-driven behavioral interventions designed to improve medication adherence among individuals with T2D. Across the included studies, a wide range of digital platforms was used to deliver adherence support, including mobile apps, SMS messaging, telemedicine systems, cloud-based platforms, conversational AI, and remote monitoring technologies. Most studies reported improvements in medication adherence, and many also observed improvements in glycemic outcomes. These findings suggest that digital health technologies are becoming an increasingly important component of contemporary diabetes care by extending medication adherence support beyond conventional face-to-face clinical encounters.

The growing adoption of digital interventions also reflects a broader transition toward more patient-centered and continuous models of diabetes management. Advances in mobile connectivity, telehealth infrastructure, wearable devices, and remote monitoring technologies have created new opportunities to deliver behavioral support outside traditional clinical settings [66]. Digital platforms allow individuals with T2D to access educational resources, receive medication reminders, and communicate with healthcare providers in real time, thereby improving continuity of care. Rather than functioning solely as reminder systems, these technologies increasingly serve as integrated platforms that support multiple aspects of diabetes self-management, including medication use, symptom monitoring, education, and clinical communication. Because effective diabetes management requires sustained medication use and long-term self-management behaviors, scalable digital health tools may complement routine clinical care by overcoming barriers related to geographic distance, time constraints, and limited healthcare resources [48].

Our findings are consistent with recent systematic reviews showing that digital health interventions can improve medication adherence and may also contribute to better glycemic control in individuals with T2D [67]. Building on this evidence, the present review extends previous work by focusing specifically on digital adherence interventions published since 2021 and by characterizing the behavioral strategies embedded within contemporary technologies. Rather than identifying a single superior technology, the evidence suggests that interventions are most effective when they integrate complementary behavioral strategies to address the multiple determinants of medication adherence. This observation provides an important foundation for understanding the behavioral mechanisms that underpin successful digital adherence interventions.

### Behavioral Mechanisms and Integrated Intervention Design

One of the principal findings of this review is that successful digital adherence interventions rarely relied on a single behavioral strategy. Instead, most interventions incorporated multiple complementary components, including medication reminders, educational content, self-monitoring tools, patient-provider communication, and peer support [41, 47, 48, 51, 54, 57]. This pattern reflects the multidimensional nature of medication adherence in T2D, which is influenced by interacting cognitive, behavioral, emotional, and contextual factors rather than forgetfulness alone [45, 68]. Patients may intentionally alter medication use due to concerns about treatment necessity or adverse effects, while unintentional nonadherence may arise from competing daily responsibilities, complex medication regimens, or difficulty integrating medications into everyday routines [69]. Interventions that simultaneously address several of these barriers are therefore more likely to provide meaningful and sustained adherence support than those targeting a single determinant.

Medication reminders remain one of the most frequently incorporated intervention components and appear to play an important role in supporting the implementation phase of prescribed medication regimens by reducing missed doses associated with forgetfulness [18, 40]. However, reminders alone are unlikely to address the diverse reasons underlying nonadherence. For this reason, many interventions integrated reminders with educational resources delivered through videos, infographics, interactive learning modules, or digital coaching [47, 57]. These educational strategies may strengthen patients’ understanding of diabetes management, reinforce perceptions of medication necessity, and address concerns regarding treatment effectiveness or adverse effects [47, 57, 70]. Several interventions further incorporated interactive learning activities, progress tracking, or gamification [16, 38], which may further strengthen self-efficacy, maintain motivation, and encourage sustained medication-taking behaviors.

Beyond reminders and education, self-monitoring and personalized feedback represented another recurring behavioral mechanism. Many digital platforms enabled individuals to record medication use, blood glucose levels, or other health indicators while providing tailored feedback based on patient-reported or remotely monitored data [41, 51]. These features may encourage self-reflection, reinforce positive behaviors, and facilitate timely adjustments to self-management strategies. Personalized feedback also aligns with behavior change principles by allowing interventions to adapt to patients’ changing needs instead of providing static educational information [16, 49].

Many interventions also strengthened communication between patients and healthcare professionals. Secure messaging, teleconsultations, remote monitoring, and automated data sharing enable healthcare professionals to monitor patient progress and provide individualized feedback or treatment recommendations [41, 48, 51, 61, 63]. Beyond supporting clinical monitoring, these communication channels strengthen therapeutic relationships, support shared decision-making, and allow concerns about medications or disease management to be addressed before they become barriers to adherence. Early identification of suboptimal adherence or deteriorating glycemic control may also create opportunities for more timely clinical intervention and treatment adjustment [71].

Several interventions additionally incorporated peer support features through online communities, discussion forums, or messaging platforms. These features allowed individuals with T2D to exchange experiences, discuss practical strategies for self-management, and provide mutual encouragement [16, 59]. Peer interaction may reduce feelings of isolation, enhance motivation, and reinforce confidence in self-management through social learning and shared experiences. Although peer-support functions were less frequently incorporated than reminders or educational components, they provide an additional source of emotional and practical support that may complement professional care and promote sustained engagement with digital interventions [72].

Across the included studies, the evidence suggests that intervention effectiveness depends less on the specific technology being used than on how behavioral strategies are combined to address multiple adherence barriers. Interventions reporting improvements in medication adherence and glycemic outcomes were more likely to combine reminders, education, self-monitoring, personalized feedback, and ongoing communication than to rely on any single digital function. This finding is consistent with contemporary models of medication adherence, which emphasize that adherence is shaped by interacting behavioral, psychological, and contextual determinants rather than a single cause [2].

Although combining multiple behavioral strategies may enhance medication adherence, these comprehensive interventions can also increase treatment burden and patient workload. Multifaceted digital programs frequently require users to complete repeated self-monitoring, review educational materials, respond to reminders, communicate with healthcare professionals, and navigate multiple digital functions [73]. These activities require time, attention, digital literacy, and sustained engagement, which may be challenging for some individuals, particularly older adults or those with limited experience using digital technologies [74]. Simply adding more intervention features is unlikely to improve outcomes if the program becomes difficult to incorporate into everyday life. Instead, future interventions should emphasize user-centered design by balancing comprehensiveness with simplicity, minimizing unnecessary workload, and tailoring intervention intensity to patients’ individual needs, preferences, and capabilities.

### Implementation Considerations for Sustainable Digital Adherence Interventions

Although most included studies reported improvements in medication adherence, intervention effectiveness varied across study settings and populations. Some studies reported improvements in glycemic control without corresponding changes in medication adherence [58, 61, 64, 66, 67], while others observed improved adherence without measurable changes in glycemic outcomes [4, 38, 49, 53, 56, 65]. These mixed findings likely reflect differences in intervention design, participant characteristics, baseline adherence, follow-up duration, and outcome measurement methods, which make direct comparisons across studies challenging. Moreover, improvements in medication-taking behavior do not necessarily translate into immediate improvements in metabolic outcomes. Glycemic control is influenced by numerous factors beyond medication adherence, including dietary behaviors, physical activity, treatment intensification, and underlying disease progression [1]. Meaningful improvements in glycemic control may also require sustained behavioral changes over longer follow-up periods before becoming clinically apparent.

The findings of this review also indicate that successful implementation depends not only on intervention design but also on patient engagement and the broader context in which digital interventions are delivered. Tailoring interventions to individual patient needs appears to be critical, as generic educational messages or standardized reminders may not adequately address specific adherence barriers [38, 75]. Individuals with T2D experience diverse challenges related to medication beliefs, treatment complexity, self-management capacity, and daily routines. Personalized interventions that adapt educational content, reminder schedules, or behavioral feedback according to individual circumstances may therefore be better positioned to support long-term adherence than standardized approaches.

Intervention success also depends on whether individuals continue to engage with digital technologies over time. The effectiveness of digital adherence programs depends not only on the behavioral strategies they incorporate but also on whether individuals actively use these features over time [76]. Although intervention content was generally well described across the included studies, reporting of implementation outcomes was considerably less consistent. Measures such as login frequency, response rates to reminders, duration of platform use, feature utilization, and engagement trajectories were frequently absent or incompletely reported. This limited the ability to determine whether improvements in medication adherence were driven primarily by the intervention itself or by participants’ sustained engagement with the technology. Future studies should routinely evaluate implementation outcomes alongside clinical effectiveness to identify which intervention components are most acceptable, sustainable, and influential in supporting long-term medication adherence.

Digital literacy, technology access, and usability of digital platforms may influence engagement with and effectiveness of these interventions. Older adults and individuals with limited digital experience may encounter greater challenges when interacting with complex digital platforms, which potentially reduces sustained engagement [77]. These findings highlight the importance of designing digital tools that are intuitive, accessible, and adaptable to the diverse digital capabilities of individuals with T2D.

Implementation should also be considered within the broader social and healthcare context. Social determinants of health, including socioeconomic status, education attainment, access to healthcare services, financial resources, and digital infrastructure, can affect both medication adherence and engagement with digital health technologies. Individuals with limited internet access, lower health literacy, or restricted access to healthcare services may derive fewer benefits from digital interventions despite having greater clinical need [78]. Integrating digital adherence programs with healthcare systems and community resources may help address these barriers by improving access to multidisciplinary care, financial assistance, culturally appropriate education, and ongoing clinical support [79]. Ultimately, addressing these structural determinants will be essential to ensure that digital health technologies reduce, rather than inadvertently widen, existing disparities in diabetes care.

### Artificial Intelligence and Future Directions

Artificial intelligence represents one of the most rapidly evolving areas of digital diabetes care, although evidence supporting its role in medication adherence remains limited. Only one included study evaluated a voice-based conversational AI intervention [52]; therefore, the available evidence is insufficient to determine whether AI-enabled interventions provide advantages over existing digital approaches for improving medication adherence in T2D.

Despite the limited clinical evidence available, AI has the potential to transform digital adherence support from static educational platforms into adaptive and personalized systems. By integrating real-time physiological data, medication-taking behaviors, and patient-reported outcomes, AI may enable interventions to identify adherence patterns, predict periods of elevated nonadherence risk, and deliver tailored behavioral support precisely when it is needed [80, 81]. Conversational agents supported by natural language processing may further enhance patient engagement by providing interactive education, answering medication-related questions, reinforcing self-management behaviors, and supporting shared decision-making through more personalized communication [82]. However, realizing this potential will require more than technological innovation alone. Future research should evaluate not only the clinical effectiveness of AI-enabled interventions but also their implementation outcomes, patient acceptability, cost-effectiveness, and integration into routine healthcare delivery. In addition, challenges related to data privacy, algorithmic transparency, bias, clinical accountability, and regulatory oversight must be carefully addressed before AI-enabled technologies can be widely adopted in diabetes care.

To advance the field, future research should move beyond evaluating individual technologies toward identifying the optimal combinations of behavioral components that can be integrated into sustainable and person-centered digital care models. Greater attention should also be given to understanding how intervention intensity, treatment burden, user engagement, and healthcare system integration interact to influence long-term medication adherence. As digital health technologies continue to evolve, implementation research will be essential to determine how integrated digital and AI-enabled interventions can be delivered equitably, incorporated into routine clinical workflows, and sustained across diverse healthcare settings.

### Strengths and Limitations

This scoping review provides an updated overview of recent digital health interventions designed to improve medication adherence among individuals with T2D. By focusing on studies published between 2021 and 2025, the review captures emerging technological approaches and evolving intervention strategies in the rapidly expanding field of digital health. In addition to describing intervention characteristics, this review synthesizes recurring behavioral strategies across diverse digital technologies and highlights potential mechanisms through which these approaches may improve medication adherence. These findings may inform the development of more integrated, person-centered, and scalable digital adherence interventions for future diabetes care.

Several limitations should be considered when interpreting the findings of this review. As a scoping review, the primary objective was to map existing interventions rather than to evaluate their effectiveness quantitatively [32]. Accordingly, formal assessments of methodological quality or risk of bias assessments and meta-analytic synthesis were not conducted. Therefore, the findings should be interpreted as a descriptive overview of the available evidence rather than as evidence of comparative intervention effectiveness. The review was restricted to studies published in English between 2021 and 2025, which may have excluded relevant research reported in other languages or earlier publications in this rapidly evolving field. In addition, considerable heterogeneity in intervention designs, digital platforms, study populations, follow-up duration, and outcome measures limited direct comparisons across studies.

Another important limitation relates to the inconsistent reporting of participant engagement and intervention utilization. Measures such as login frequency, response rates to reminders, duration of platform use, adherence to intervention activities, and utilization of individual intervention features were frequently incomplete or absent. As a result, it was difficult to distinguish whether intervention effectiveness reflected the behavioral strategies themselves or participants’ sustained engagement with the digital technologies. Routine reporting of implementation outcomes, including engagement metrics, intervention fidelity, usability, and feature-specific utilization, would strengthen future evaluations and facilitate comparisons across digital adherence interventions.

Finally, although this review identified several emerging technologies, including conversational AI and remote monitoring systems, evidence supporting these approaches remains limited. As digital health technologies continue to evolve rapidly, ongoing evidence synthesis will be necessary to evaluate their effectiveness, implementation, and long-term sustainability in routine diabetes care.

## Conclusions

Digital health technologies are playing an increasingly important role in supporting medication adherence among individuals with T2D and show considerable promise for improving both medication-taking behaviors and broader diabetes self-management. The evidence synthesized in this review suggests that intervention effectiveness depends less on the specific digital platform than on the integration of complementary behavioral strategies that address the multifactorial determinants of medication adherence. To achieve these benefits, successful implementation requires sustained user engagement, equitable access, seamless integration into routine healthcare delivery, and careful consideration of treatment burden to ensure that interventions remain accessible and acceptable for diverse patient populations. Future research should focus on identifying the most effective combinations of behavioral components, optimizing person-centered intervention design, and generating high-quality evidence to support implementation in real-world clinical settings. As AI-enabled technologies and other advanced digital health tools continue to evolve, rigorous evaluation of their clinical effectiveness, implementation, safety, equity, and long-term sustainability will be essential to facilitate their integration into routine diabetes care and maximize their potential to improve long-term health outcomes. 

## Supplementary Information

Below is the link to the electronic supplementary material.

Supplementary Material 1.

## Data Availability

No datasets were generated or analysed during the current study.

## Appendix A. Literature Search Strategy for PubMed, Scopus, and Web of Science

### Search term for PubMed: 326 records

((type 2 diabetes[Title/Abstract]) AND (((intervention[Title/Abstract]) OR (education[Title/Abstract])) OR (program[Title/Abstract]))) AND ((((medication adherence[Title/Abstract]) OR (medicine adherence[Title/Abstract])) OR (medication compliance[Title/Abstract])) OR (medicine compliance[Title/Abstract])) AND (2021:2025[pdat])

### Search term for Scopus: 1276 records

(TITLE-ABS-KEY (type 2 diabetes)) AND ((TITLE-ABS-KEY (intervention) OR TITLE-ABS-KEY (education) OR TITLE-ABS-KEY (program))) AND ((TITLE-ABS-KEY (medication adherence) OR TITLE-ABS-KEY (medicine adherence) OR TITLE-ABS-KEY (medication compliance) OR TITLE-ABS-KEY (medicine compliance))) AND PUBYEAR > 2020 AND PUBYEAR < 2026.

### Search term for Web of Science: 634 records

(((AB=(type 2 diabetes)) AND AB=(intervention or education or program)) AND AB=(medication adherence or medicine adherence or medication compliance or medicine compliance)) AND PY=(2021–2025).

## Key References

- Nelson LA, Greevy RA, Spieker A, Wallston KA, Elasy TA, Kripalani S, et al. Effects of a tailored text messaging intervention among diverse adults with type 2 diabetes: evidence from the 15-month REACH randomized controlled trial. Diabetes Care. 2021;44(1):26-34. doi: 10.2337/dc20-0961
  - A 15-month randomized controlled trial evaluated a tailored text-messaging intervention for diabetes self-management. Participants receiving interactive and adherence-focused messages showed modest A1C reductions at 6 months, and larger reductions were observed among those with poorer baseline glycemic control. The intervention also improved medication adherence and diet through 12 months and self-efficacy through 6 months. Despite high engagement, the effects were not sustained at 15 months. This finding indicates that text messaging alone may be insufficient for long-term diabetes control.
- Bartlett YK, Farmer A, Newhouse N, Miles L, Kenning C, French DP. Effects of using a text message intervention on psychological constructs and the association between changes to psychological constructs and medication adherence in people with type 2 diabetes: results from a randomized controlled feasibility study. JMIR Form Res. 2022;6(4):e30058. doi: 10.2196/30058
  - A 6-month randomized feasibility trial evaluated a theory-based text-messaging intervention to improve oral medication adherence in adults with T2D. Messages incorporated behavior change techniques targeting psychological determinants of adherence. Compared with usual care, the intervention improved several adherence-related constructs, including necessity beliefs, intention, self-efficacy, action control, prompts and cues, social support, and satisfaction with consequences. Changes in intention, self-efficacy, automaticity, and satisfaction were positively associated with improvements in self-reported medication adherence. This result suggests that text-messaging interventions may enhance adherence by targeting key psychological mechanisms.
- Nayak A, Vakili S, Nayak K, Nikolov M, Chiu M, Sosseinheimer P, et al. Use of voice-based conversational artificial intelligence for basal insulin prescription management among patients with type 2 diabetes: A randomized clinical trial. JAMA Netw Open. 2023;6(12):e2340232. doi: 10.1001/jamanetworkopen.2023.40232
  - A randomized clinical trial evaluated a voice-based conversational artificial intelligence (AI) application for home basal insulin titration in adults with T2D over 8 weeks. Compared with standard care, the AI intervention enabled faster achievement of optimal insulin dosing, improved insulin adherence, and increased rates of glycemic control. Participants in the AI group also showed greater reductions in fasting glucose and diabetes-related emotional distress. These findings indicate that voice-based AI may support insulin titration and improve diabetes management outcomes.
- Caballero Mateos I, Morales Portillo C, Lainez López M, Vilches-Arenas Á. Efficacy of a digital educational intervention for patients with type 2 diabetes mellitus: Multicenter, randomized, prospective, 6-month follow-up study. J Med Internet Res. 2025;27:e60758. doi: 10.2196/60758
  - A multicenter randomized study evaluated a 6-month digital educational intervention delivered through social networks and digital tools with support from a “Digital Coach” in adults with T2D initiating glucagon-like peptide-1 receptor agonists. Compared with usual care, the intervention produced greater reductions in A1C, fasting glucose, body weight, and body mass index. It also improved diabetes knowledge, medication adherence, patient experience, and quality-of-life satisfaction. These findings suggest that digitally delivered education and coaching can improve metabolic control and self-management in people with T2D.
