# Supplementary material for: A Scoping Review of Digital Behavioral Approaches to Improve Medication Adherence in Type 2 Diabetes
Source: Curr Diab Rep. 2026 Sep 5;26(1):27. doi: 10.1007/s11892-026-01642-5 (PMC13546315; doi:10.1007/s11892-026-01642-5)
Supplement: Supplementary file 1 [file 11892_2026_1642_MOESM1_ESM.pdf]

**Appendix 1. Literature search strategy for PubMed, Scopus, and Web of Science**

**Search term for PubMed: 326 records**

((type 2 diabetes[Title/Abstract]) AND (((intervention[Title/Abstract]) OR (education[Title/Abstract])) OR (program[Title/Abstract]))) AND ((((medication adherence[Title/Abstract]) OR (medicine adherence[Title/Abstract])) OR (medication compliance[Title/Abstract])) OR (medicine compliance[Title/Abstract])) AND (2021:2025[pdat])

**Search term for Scopus: 1276 records**

(TITLE-ABS-KEY (type 2 diabetes)) AND ((TITLE-ABS-KEY (intervention) OR TITLE-ABS-KEY (education) OR TITLE-ABS-KEY (program))) AND ((TITLE-ABS-KEY (medication adherence) OR TITLE-ABS-KEY (medicine adherence) OR TITLE-ABS-KEY (medication compliance) OR TITLE-ABS-KEY (medicine compliance))) AND PUBYEAR > 2020 AND PUBYEAR < 2026

**Search term for Web of Science: 634 records**

(((AB=(type 2 diabetes)) AND AB=(intervention or education or program)) AND AB=(medication adherence or medicine adherence or medication compliance or medicine compliance)) AND PY=(2021-2025)
